# Targeting the YAP/TAZ Hippo signaling pathway for oral cancer treatment

**DOI:** 10.17179/excli2024-7652

**Published:** 2024-08-26

**Authors:** Muthu Thiruvengadam

**Affiliations:** 1 Department of Applied Bioscience, College of Life and Environmental Science, Konkuk University, Seoul 05029, Republic of Korea; 2 Center for Global Health Research, Saveetha Medical College & Hospitals, Saveetha Institute of Medical and Technical Sciences (SIMATS), Thandalam, Chennai, 602 105, Tamil Nadu, India

## ⁯⁯⁯

Oral cancer is among the six most prevalent cancers globally. Its prognosis is often aggravated by delayed treatment, which diminishes the survival rates. The Hippo pathway assists as a critical regulator, exerting negative control over the activity of its downstream effector, the YAP, and its homolog transcriptional co-activator with PDZ-binding motif (TAZ). YAP/TAZ plays a vital role in cell proliferation, survival, and stem cell maintenance during embryonic development. The diminished activation of YAP/TAZ results in developmental abnormalities, impaired repair mechanisms, and organ shrinkage. On the contrary, inactivation of the Hippo pathway leads to nuclear translocation of YAP/TAZ, which increases cell proliferation and tumorigenesis. Notably, dysregulation of YAP and TAZ fuels protumorigenic signaling in oral squamous cell carcinoma. We addressed the manifold functions of YAP and TAZ in oral cancer.

The YAP/TAZ Hippo signaling pathway presents a promising avenue for oral cancer therapy because of its critical involvement in regulating cellular proliferation, survival, and tissue homeostasis. Its dysregulation significantly promotes the growth and progression of oral cancer (Zinatizadeh et al., 2019[9]). The therapeutic potential of targeting this pathway revolves around its intricate signaling processes. Initially, various upstream factors, such as cell-cell contact, mechanical stimuli, and soluble molecules, influence the core Hippo kinase cascade activity. This cascade comprises MST1/2 and LATS1/2 kinases, which phosphorylate and inhibit the transcriptional coactivators YAP and TAZ. When unphosphorylated, YAP/TAZ translocates to the nucleus, where it interacts with transcription factors, such as TEAD, promoting the expression of genes linked to proliferation and survival. Strategies for targeting the YAP/TAZ pathway in oral cancer therapy include diverse approaches (Zinatizadeh et al., 2019[9]). Small-molecule inhibitors or antibodies can disrupt crucial protein-protein interactions necessary for YAP/TAZ nuclear localization or their interaction with transcription factors. Additionally, upstream regulators or downstream effectors of YAP/TAZ signaling are potential therapeutic targets (Zinatizadeh et al., 2019[9]). These interventions aim to curb aberrant YAP/TAZ activity, thereby impeding oral cancer cell proliferation, inducing apoptosis, and potentially sensitizing tumors to conventional treatments, such as chemotherapy and radiotherapy. Thus, the comprehension and manipulation of the YAP/TAZ Hippo signaling pathway offer hope for the growth of new oral cancer therapies characterized by improved efficacy and reduced side effects.

Patients with cancers overexpressing YAP exhibit poorer overall survival rates than those without YAP expression. Elevated nuclear levels of YAP/TAZ have been linked to the development of resistance to chemotherapy in OSCC treated with cisplatin and cetuximab. Nuclear accumulation of YAP/TAZ is evident in hyperplastic, dysplastic, and high-grade OSCC (Wei et al., 2013[8]). In both *in vitro *and* in vivo* studies, YAP and TAZ knockdown reduced proliferation increased the expression of pro-apoptotic genes, and inhibited primary tumor growth. Nuclear YAP/TAZ facilitates Wnt-induced signaling, which is crucial for OSCC progression. Upon nuclear translocation, YAP/TAZ binds to the TEAD transcription factor and promotes the transcription of genes important for cell proliferation. Notably, YAP/TAZ induced the expression of *TEAD4*, which was considerably improved in advanced stages and grades of OSCC. *TEAD4* contributes to TAZ nuclear accumulation through a feed-forward mechanism that promotes oncogenic transformation. YAP/TAZ complexes with DNA-binding proteins such as *SNAIL*, *SLUG*, *AP-1*, *Myc*, and *NICD/RBPJ* regulate gene expression, as they cannot directly bind to DNA. YAP transcriptionally activates genes including *BIRC5/survivin*, *BIRC2/cIAP1*, *FOS*, *c-myc*, *SOX4*, *CYR61*, and *CTGF,* and upregulates *PIEZO1*, a calcium channel driving OSCC cell proliferation (Lopez-Hernandez et al., 2021[6]). YAP's proliferative potential of YAP involves inducing the transcription of BIRC5/survivin and inhibiting CDKN1A/p21 transcription.

MicroRNA-30c modulates the expression of MAML1/2 proteins, thereby enhancing the oncogenic action of YAP/TAZ by promoting their activation in cancer patients. TIMP-1 induces YAP/TAZ activation through the TIMP-1-CD63-Integrin β1 axis, leading to LATS1/2 inactivation via Src activation and RhoA-mediated F-actin assembly. LATS1/2 inactivation stabilizes and translocates YAP/TAZ to the nucleus, resulting in CTGF synthesis and increased cancer cell proliferation (Ando et al., 2018[1]). Microbial dysbiosis, particularly that involving *Porphyromonas gingivalis* in the digestive tract, increases the risk of ESCC. *P. gingivalis* promotes ESCC cell growth, migration, and metastasis through TGF-β, activating the Smads/YAP/TAZ/TEAD1 complex and initiating downstream target gene expression, ultimately inducing epithelial-mesenchymal transition and cancer stemness (Baima et al., 2023[2]). Viral GPCR inhibits the Hippo pathway kinases LATS1/2 via Gq/11 and G12/13, thereby activating YAP/TAZ and driving cancer progression (Hsu et al., 2015[4]).

*TAZ* overexpression induces the transformation of non-cancer stem cells into cancer stem cells by promoting EMT and chemoresistance in OSCC cells. Nuclear localization of YAP correlates with cisplatin resistance in oral cancer cells, while studies employing siRNA knockdown have demonstrated that inhibiting YAP enhances sensitivity to cisplatin in OSCC parental cell lines. *YAP* expression in cancer is associated with immunosuppression through increased *PD-L1* expression, leading to the suppression of cytotoxic lymphocyte activity and reduced cytokine production (Kim et al., 2018[5]). VEGF maintains cancer stemness by stimulating sustained angiogenesis in cancer cells via TAZ activation (Elaimy et al., 2019[3]). Along with various kinases, ECM rigidity regulates YAP/TAZ localization in the cytoplasm or nucleus of cancer cells. Soft ECM inhibits YAP/TAZ activity in the cytoplasm, whereas rigid ECM promotes nuclear translocation for gene transcription. Cancer cell contractility is enhanced by ECM interaction, transmitting signals to the F-actin cytoskeleton and activating YAP/TAZ through either Hippo pathway-dependent or independent LATS1/2 phosphorylation. YAP also participates in a feed-forward mechanism by inducing ECM remodeling, activating the actomyosin cytoskeleton, and upregulating *ANLN* and *DIAPH3* expression, thereby increasing ECM rigidity and affecting cancer patient prognosis (Matsui and Lai, 2013[7]).

In summary, the influence of YAP/TAZ extends beyond the Hippo pathway, serving as a central hub in multiple signaling pathways crucial for cancer stemness, resistance to therapies (chemoresistance and immunoresistance), and shaping of the tumor microenvironment. Consequently, directing interventions towards this multifaceted YAP/TAZ transcriptional coactivator holds promise for enhancing the survival rates of patients with oral cancer in the foreseeable future. Nonetheless, further exploration of the molecular networks and potential metabolic alterations mediated by YAP/TAZ in oral cancer is imperative.

## Abbreviations

ANLN Anillin

BIRC5 Baculoviral IAP repeat containing 5

CTGF Connective tissue growth factor

DIAPH3 Diaphanous related formin 3

ECM Extracellular matrix

EMT Epithelial-mesenchymal transition

ESCC Esophageal squamous cell carcinoma

LATS1 Large tumor suppressor kinase 1

MAML1/2 Mastermind-like 1 and 2

MST mammalian STE20-like protein kinase 1

NICD Transcription factor moiety of Notch

OSCC Oral squamous cell carcinoma

PD-L1 Programmed death-ligand 1

PDZ Post synaptic density protein (PSD95), Drosophila disc large tumor suppressor (Dlg1), and Zonula occludens-1 protein (zo-1)

PIEZO1 Piezo-type mechanosensitive ion channel component 1

RBPJ recombination signal binding protein for immunoglobulin kappa J region

SMAD Suppressor of Mothers against Decapentaplegic.

TAZ Transcriptional coactivator with PDZ-binding motif

TEAD1 TEA domain transcription factor 1

TIMP-1 TIMP metallopeptidase inhibitor 1

VEGF Vascular endothelial growth factor

YAP Yes-associated protein

## Declaration

### Funding

This study received no external funding.

### Conflict of interest

The author declares no conflicting interests. 

### Data availability

All data used are publicly available.
